# Multi‐seasonal modelling of plant‐nematode interactions reveals efficient plant resistance deployment strategies

**DOI:** 10.1111/eva.12989

**Published:** 2020-05-22

**Authors:** Samuel Nilusmas, Mathilde Mercat, Thomas Perrot, Caroline Djian‐Caporalino, Philippe Castagnone‐Sereno, Suzanne Touzeau, Vincent Calcagno, Ludovic Mailleret

**Affiliations:** ^1^ Université Côte d'Azur, INRAE, CNRS, ISA Sophia Antipolis France; ^2^ Université Côte d'Azur, INRIA, INRAE, CNRS, Sorbonne Université, BIOCORE Sophia Antipolis France

**Keywords:** computer simulation, crop protection, disease resistance, mathematical model, nematode infections, pest control, population dynamics, rotation, seasons, virulence

## Abstract

Root‐knot nematodes, *Meloidogyne spp*., are soil‐borne polyphagous pests with major impact on crop yield worldwide. Resistant crops efficiently control avirulent root‐knot nematodes, but favour the emergence of virulent forms. Since virulence is associated with fitness costs, susceptible crops counter‐select virulent root‐knot nematodes. In this study, we identify optimal rotation strategies between susceptible and resistant crops to control root‐knot nematodes and maximize crop yield. We developed an epidemiological model describing the within‐season dynamics of avirulent and virulent root‐knot nematodes on susceptible or resistant plant root‐systems, and their between‐season survival. The model was fitted to experimental data and used to predict yield‐maximizing rotation strategies, with special attention to the impact of epidemic severity and genetic parameters. Crop rotations were found to be efficient under realistic parameter ranges. They were characterized by low ratios of resistant plants and were robust to parameter uncertainty. Rotations provide significant gain over resistant‐only strategies, especially under intermediate fitness costs and severe epidemic contexts. Switching from the current general deployment of resistant crops to custom rotation strategies could not only maintain or increase crop yield, but also preserve the few and valuable R‐genes available.

## 1 INTRODUCTION

As the global population increases, finding effective and durable crop protection strategies has become a major challenge (Cunniffe et al., 2015). Predictions indicate that population growth, combined with changes in dietary habits, will lead to an increase in the global food demand by at least 50% in 2050 (Springmann, Godfray, Rayner, & Scarborough, 2016; Tilman, Balzer, Hill, & Befort, 2011). To meet this demand, crop production will have to increase, with expected negative environmental impacts (biodiversity and forest loss, reduced freshwater availability, soil degradation and CO2 emissions) if relying on the extensive use of chemical pesticides and monocultures (Stoate et al., 2009; Tilman et al., 2001; Zhan, Thrall, Papaïx, Xie, & Burdon, 2015). Furthermore, crop losses are expected to increase as well, owing to the emergence or evolution of plant pests and diseases (Palumbi, 2001; Stukenbrock & McDonald, 2008). These trends call for experimental and theoretical studies aiming at protecting crops and increasing their yield durably, while reducing pesticide dependence. In this context, the development of environmentally friendly pest management strategies based on biological control, better cultural practices and the use of resistant plants are very promising (Mundt, 2014; Van Lenteren, Bolckmans, Köhl, Ravensberg, & Urbaneja, 2018; Zhan et al., 2015).

Natural plant resistance is among the most efficient alternatives to pesticides in economic, environmental and social terms. Qualitative plant resistance rests on gene‐for‐gene interactions (Flor, 1971), in which an avirulent gene (Avr‐gene) in the pest or pathogen interacts with a major resistance gene (R‐gene) in the plant, resulting in disease resistance through what is usually called effector‐triggered immunity or incompatible reaction (Dangl & Jones, 2001; Jones & Dangl, 2006). If the R‐gene is inactive or absent, or equivalently if the pest lacks the Avr‐locus, the interaction instead results in plant infection. Major R‐genes are rare in nature and plant breeders mostly work on the introgression of a small list of major R‐genes into different genetic backgrounds to create commercial crop cultivars. Therefore, farmers ultimately employ the same resistance genes over several years and on large spatial scales. Such an intensive use of resistance generates strong selection pressures on populations of avirulent pests, which can lose the Avr‐gene through mutation, causing the emergence and establishment of virulent variants (Castagnone‐Sereno, 2002; Garcia‐Arenal, Fraile, & Malpica, 2003; Leonard, 1977; McDonald & Linde, 2002; Parlevliet, 2002).

According to Johnson (1981), a durable resistance is one that remains effective in a cultivar for a long period of time despite its widespread cultivation. In a gene‐for‐gene system, resistance durability may depend on the time required for a mutation at the Avr‐gene to occur and the time for the virulent pathogen to establish (Barrett, Thrall, Burdon, & Linde, 2008; Brown, 2015; Fabre, Bruchou, Palloix, & Moury, 2009; Stuthman, Leonard, & Miller‐Garvin, 2007; Van den Bosch & Gilligan, 2003; Zhan et al., 2015). The latter might be expected to be very short, considering the huge advantage for a pathogen to overcome resistance and become virulent. However, significant polymorphism exists at virulence genes, that can at least partly be explained by fitness costs associated with virulence (Laine & Tellier, 2008; Stahl, Dwyer, Mauricio, Kreitman, & Bergelson, 1999; Tian, Traw, Chen, Kreitman, & Bergelson, 2003). Numerous studies have reported fitness costs in bacteria (Cruz et al., 2000; Leach, Vera Cruz, Bai, & Leung, 2001), oomycetes (Montarry, Hamelin, Glais, Corbière, & Andrivon, 2010) or viruses (García‐Arenal & Fraile, 2013). The existence of fitness costs implies that even though virulent pathogens are selected for in resistant crops, they are selected against in susceptible crops, where avirulent pathogens grow and reproduce faster.

Several approaches to improve the durability of resistant genes have been proposed (Fabre, Rousseau, Mailleret, & Moury, 2012, 2015; Lof & van der Werf, 2017; Papaïx, Touzeau, Monod, & Lannou, 2014; Van den Bosch & Gilligan, 2003). The most common deployment strategies are mixtures, mosaics and rotations of resistant and susceptible plant cultivars, all of which exploit spatial and/or temporal heterogeneity in selection pressures (Djidjou‐Demasse, Moury, & Fabre, 2017; Kiyosawa, 1982; Mundt, 2002; Pink, 2002; Rimbaud, Papaïx, Barrett, Burdon, & Thrall, 2018).

Root‐knot nematodes (*Meloidogyne spp*., Kofold & White) are ubiquitous plant pathogens (Jones et al., 2011; Trudgill & Blok, 2001). They are obligate extremely polyphagous plant endoparasites, which cause damage to the roots of thousands of host plant species (Perry et al., 2009; Wesemael, Viaene, & Moens, 2011). Overall, their economic impact has been estimated at over 121 billion dollars of crop losses each year (Chitwood, 2003). For several decades, controlling these parasites has relied on chemical treatments, but these proved extremely damaging to the environment and to human health and have been banned (Abad & Williamson, 2010; Zasada et al., 2010). Root‐knot nematode infestations are becoming an increasing source of concern in vegetable production due to these recent restrictions on the use of chemical nematicides. For instance, a survey by Djian‐Caporalino (2012) showed that in the South of France, more than 40% of horticultural holdings are impacted by root‐knot nematodes, sometimes with insurmountable financial consequences. The fight against root‐knot nematodes is now therefore largely based on the use of plant cultivars bearing resistance genes (Williamson & Roberts, 2009). However, resistance breaking by virulent nematodes has been demonstrated in the laboratory (Djian‐Caporalino et al., 2011; Jarquin‐Barberena, Dalmasso, de Guiran, & Cardin, 1991; Meher, Gajbhiye, Chawla, & Singh, 2009) and is more and more observed in field conditions (Verdejo‐Lucas, Cortada, Sorribas, & Ornat, 2009).

As for other plant parasites, virulence in root‐knot nematodes is associated with a fitness cost and it was shown that virulence reduces the capacity to infect the plant, as well as the number of eggs laid per female (Castagnone‐Sereno, Bongiovanni, & Wajnberg, 2007; Djian‐Caporalino et al., 2011). Therefore, setting up rotation strategies of resistant and susceptible cultivars has the potential to increase the durability of resistance genes and the efficacy of resistance‐based nematode control. However, field tests of deployment strategies in terms of epidemic control and resistance durability remain difficult, owing to their labour intensive nature and to the long time horizons involved (Djian‐Caporalino et al., 2014).

In these conditions, modelling approaches constitute a powerful way to explore resistant plant deployment strategies and assess their efficiency to reduce yield losses and increase control durability (Brown, 2015; Papaïx, Rimbaud, Burdon, Zhan, & Thrall, 2018). The literature is very poor in theoretical modelling studies addressing the control of soil‐borne pathogens with limited dispersal, such as root‐knot nematodes. For instance, most studies deal with pathogens that can disperse over large spatial scales (Djidjou‐Demasse et al., 2017; Fabre, Rousseau, Mailleret, & Moury, 2012; Gilligan, 1995; Lof & van der Werf, 2017; Otten & Gilligan, 2006; Thrall, Bever, Mihail, & Alexander, 1997). Root‐knot nematodes, in contrast, have very limited mobility in the soil, feeding and reproducing locally in the plant root system. Consequently, nematode populations barely mix and strategies based on spatial arrangements are poorly applicable. In addition, the major root‐knot nematode species reproduce solely by clonal reproduction so that techniques based on recombination between virulent and avirulent genotypes do not operate.

The purpose of this study was to assess quantitatively whether rotation strategies between susceptible and nematote‐resistant cultivars can efficiently control nematodes, and to determine which optimal crop rotation strategies should be used to maximize crop yield over several seasons. We did this by building a semi‐discrete plant epidemic model (Fabre et al., 2012; Mailleret, Castel, Montarry, & Hamelin, 2012; Mailleret & Lemesle, 2009), tailored to the root‐knot nematode pathosystem. The model describes the within‐season dynamics of the interaction between a plant root system and root‐knot nematodes, the overwintering dynamics between consecutive seasons and the potential evolution of the nematode population from avirulent to virulent forms. The model was parameterized from the literature and fitted to experimental data (Ehwaeti, Phillips, & Trudgill, 1998). We used the model to compute optimal crop rotation strategies with respect to a proxy of crop yield over different time horizons. Given that the fitness costs vary among R‐genes and nematode strains and are crucial to the durability of R‐genes, we paid special attention to the influence of these genetic parameters. We evaluated to what extent crop rotation provided better crop yield than the widely used resistant plant‐only strategy (pure resistant strategy) for different epidemic scenarios and genetic parameters. We also tested the robustness of our results to determine whether the effectiveness of optimal periodic rotations can be maintained even if epidemiological and genetic parameters are not known precisely. We investigated the key factors to be taken into account for optimal resistance plant deployment strategies against root‐knot nematodes.

## 2 MATERIALS AND METHODS

We developed and applied a model of the interaction between crop plants and a nematode pest. Plants can be either resistant or susceptible, and the nematodes can be either virulent or avirulent. Resistant plants do not get infected by the avirulent nematodes but they do prompt the evolution of virulent nematodes. Thus, there is a potential trade‐off. In the short‐term, resistant plants give higher yields because they are not infected by the nematodes. However, in the longer term, strategies that include susceptible plants can give higher yields by keeping the levels of virulence in the nematodes lower. We used the model, parameterized to a real‐world system of economic importance, to investigate the trade‐offs between these strategies. Furthermore, we studied the influence of parameter values, by implementing contrasted epidemic scenarios and performing a robustness analysis.

### 2.1 Study system

We focused on root‐knot nematodes of the species *Meloidogyne incognita* (Kofold & White). These are obligate endoparasites of plant roots and reproduce only by clonal reproduction. *M. incognita* is one of the most prevalent species in the warm conditions of Mediterranean countries, especially in protected crops (Wesemael et al., 2011). It is one of the most serious concerns for tomato growers in the South of France and other Mediterranean countries, for instance in Morocco where tomatoes are still planted for many consecutive years.

**FIGURE 1:**
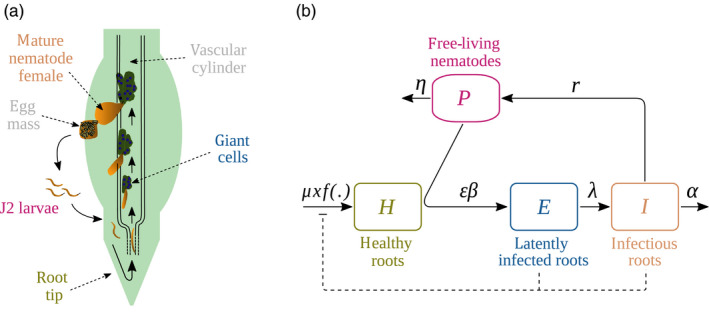
(a) Life cycle of root‐knot nematodes (adapted from Williamson and Gleason (2003) and Abad and Williamson (2010)). (b) Schematic description of model (Equation 1). Root‐knot nematode eggs hatch as J2 larvae (free‐living nematodes *P*) which can penetrate healthy parts of plant roots (healthy roots *H*). After infection, the larva migrates down to the root tip, enters the vascular cylinder and migrates up the root to settle and induce a feeding site on host cells (giant cells, latently infected roots *E*). The nematode ingests the cytoplasm of the giant cells to maturate into a pear‐shaped mature female that releases its eggs onto the root surface (infectious roots *I*) in a protective matrix (egg mass). When conditions are favourable, eggs hatch and the cycle starts again. Text colours match between both panels

The life cycle of *M. incognita* consists in four stages that can be achieved in 3–5 weeks, depending on environmental conditions (Abad & Williamson, 2010). Second‐stage juveniles dwell in the soil and penetrate the plant when a root grows in their vicinity. Once a nematode reaches the vascular cylinder of the root, salivary secretions induce the creation of a feeding site. These are composed of 5–6 hypertrophied plant cells, known as giant cells. The nematode spends the rest of its life in this feeding site, where it develops until reproduction. When mature, adult females release several hundreds of eggs (between 300 and 2,000 eggs/female on average) outside the root, which will hatch into free‐living juveniles and complete the cycle (Figure 1a).

In the *Solanaceae* plant family, a few resistance genes are known to block the development and reproduction of root‐knot nematodes: the *Mi‐1* gene in tomato (Lycopersicon lycopersicum, Linnaeus) (Milligan et al., 1998) and the *N*, *Me‐1* and *Me‐3* genes in sweet pepper (*Capsicum annuum*, Linnaeus) (Djian‐Caporalino et al., 2007, 2011). The most pervasive resistance breakdown issue consists in the *Mi‐1* gene being overcome by *M. incognita* (Ornat, Verdejo‐Lucas, & Sorribas, 2001; Seid, Fininsa, Mekete, Decraemer, & Wesemael, 2015). *Mi‐1*, originally from the wild species *Solanum peruvianum*, was introgressed into tomato by interspecific crosses in the early 1940s. The first resistant varieties appeared on the market by the end of that decade. Since then, many resistant varieties have been globally deployed, all bearing the same resistance gene. Nowadays, resistance breaking by *M. incognita* populations is recorded worldwide, in virtually every area growing tomatoes (Seid et al., 2015). In this study, we will thus use *Mi‐1*/tomato/*M. incognita* as our example system.

### 2.2 Model of plant‐nematode interactions

The interaction between nematodes and plants during a cropping season was modelled as an epidemic of free‐living pests infesting and spreading among the plant root system (Figure 1b). We first consider only avirulent nematodes and a susceptible plant. The model describes in continuous time the changes in four variables: the density of free‐living nematodes in the soil (*P*_a_), the density of healthy susceptible plant roots (*H* ^S^) and the density of latent (*E*_a_) and infectious (*I*_a_) feeding sites induced by nematodes. It is represented by the following system of differential equations:

(1) $$\left\{\begin{array}{c} {\dot{P}}_{a}\;=\;-\beta P_{a}H^{s}\;-\;\eta P_{a}\;+\;rI_{a},\\ {\dot{H}}^{s}\;=\;\mu xf\;\left(H^{s},E_{a},I_{a}\right)\;-\;\varepsilon_{a}^{S}\beta P_{a}H^{S},\\ {\dot{E}}_{a}\;=\;\varepsilon_{a}^{S}\beta P_{a}H^{S}-\;\lambda E_{a},\\ {\dot{I}}_{a}\;=\;\lambda E_{a}\;-\;\alpha I_{a}. \end{array}\right.$$

When a free‐living avirulent nematode *P*_a_ comes into contact with a portion of healthy plant root *H* ^S^, the latter becomes latently infected *E*_a_ at rate $\varepsilon_{a}^{S}\beta P_{a}H^{S}$ , where *β* is the infection rate and $\varepsilon_{a}^{S}\;=\;1$ is a conversion factor between nematode and root densities (Table 1). After a time period 1/*λ*, the infected root portion becomes infectious (*I*_a_) and starts producing free‐living avirulent nematodes (*P*_a_) at rate *r*. Free‐living nematodes in the soil and infectious nematodes in the roots die at rates *η* and *α*, respectively. Roots are assumed to grow linearly with time at basic rate *µx* (Leskovar, Cantliffe, & Stoffella, 1990), where *x* is a conversion factor between root biomass and root density. Root infection by nematodes is known to impact root growth (Zeck, 1971), which is taken into account through function *f*(.). This function discounts the basic growth rate by a decreasing exponential function of infection prevalence $\pi \;=\;\frac{E_{a}\;+\;I_{a}}{H^{S}\;+\;E_{a}\;+\;I_{a}}$ multiplied by a scaling factor *k*: $f\left(H^{S},E_{a},I_{a}\right)\;=\;e^{-k\pi }$ .

**TABLE 1:** Model variables and parameters

Symbol	Description	Value(s)		Unit	Ref.
*H^X^*	Density of healthy plant root			UR	
*P*	Density of free‐living nematodes			UN	
*E*	Density of latently infected feeding sites			UR	
*I*	Density of infectious feeding sites			UR	
*H*_0_	Initial root density	6	(4.2, 7.8)	UR	[1]
*P*_0_	Initial nematode density in the soil	0.8	(0.03, 20)	UN	[2]
*p*_v_	Initial proportion of virulent nematodes	10^−3^	(7 × 10^−4^; 1.3 × 10^−3^)	–	[3]
*β*	Infection rate	1.11 × 10^−4^	(7.78 × 10^−5^, 1.44 × 10^−4^)	UR^−1^ day^−1^	[*]
*w_β_*	Fitness cost on infectiveness	0.09	(0.06, 0.12)	–	[4]
*λ*	Transition rate from *E* to *I*	0.06	(0.042, 0.078)	day^−1^	[4,5]
*r*	Nematode reproduction rate	17	(11.9, 22.1)	UN UR^−1^ day^−1^	[4]
*w_r_*	Fitness cost on reproduction	0.31	(0.22, 0.40)	–	[4]
*δ*	Fraction of virulent offspring	10^−6^	7 × 10^−7^, 1.3 × 10^−6^	–	[3]
*α*	Nematode mortality rate in roots	0.125	(0.0875, 0.1625)	day^−1^	[4,5]
*η*	Nematode mortality rate in the soil	0.04	(0.028, 0.052)	day^−1^	[6]
*φ*	Between‐season survival probability	0.4	(0.28, 0.52)	–	[7]
$\varepsilon_{y}^{X}$	Nematode infection success	0 if *X* = *R* and *y* = *a* 1 otherwise		UR UN^−1^	
*µx*	Plant root growth rate	0.315	(022, 0.41)	UR day^−1^	[1*]
*k*	Nematode impact on root growth	10.33	(7.23, 13.43)	–	[*]
τ	Duration of a cropping season	135		days	[8]

Note

All parameters, except $\varepsilon_{y}^{X}$ and *τ*, were varied for the sensitivity and robustness analyses: default value and ±30% variations (indicated in brackets) were tested; larger variations were tested for *P*_0_, in line with Ehwaeti et al. (1998).

Units: UR, number of feeding sites per gram of soil; UN, number of nematodes per gram of soil.

Sources: [1] Leskovar et al. (1990); [2] Ehwaeti et al. (1998); [3] Ploeg and Maris (1999); [4] Castagnone‐Sereno et al. (2007); [5] Ekanayake and Vito (1986); [6] Tsai (2008); [7] Castagnone‐Sereno, P., unpublished data; [8] Djian‐Caporalino, C., unpublished data; [8] Djian‐Caporalino, C., unpublished data; [*] Estimated.

The model (Equation 1) is readily extended to take into account susceptible and resistant plants, as well as the co‐occurence of avirulent and virulent nematodes. Variable *P*_v_ represents the density of virulent free‐living nematodes in the soil; similarly *I*_v_ and *E*_v_ represent the densities of feeding sites infected by latent and infectious virulent nematodes, respectively. In what follows, superscript *X* indicates the type of cultivated plant in the current cropping season, that is either susceptible (*X* = *S*) or resistant (*X* = *R*). The model, represented graphically in Figure S1, then reads:

(2) $$\left\{\begin{array}{c} {\dot{P}}_{a}\;=\;-\beta P_{a}H^{X}\;-\;\eta P_{a}+\;\left(1-\delta \right)rI_{a},\\ {\dot{P}}_{v}\;=\;-\beta P_{v}H^{X}\;-\;\eta P_{v}\;+\;\delta rI_{a}\;+\;\left(1\;-\;w_{r}\right)rI_{v},\\ {\dot{H}}^{X}\;=\;\mu xf\left(H^{X},E_{a}\;+\;E_{v},I_{a}\;+\;I_{v}\right)\;-\;\varepsilon_{a}^{X}\beta P_{a}H^{X}\;-\;\left(1-w_{\beta }\right)\;\varepsilon_{v}^{X}\beta P_{v}H^{X},\\ {\dot{E}}_{a}\;=\;\varepsilon_{a}^{X}\beta P_{a}H^{X}\;-\;\lambda E_{a},\\ {\dot{E}}_{v}\;=\;\left(1-w_{\beta }\right)\varepsilon_{v}^{X}\beta P_{v}H^{X}\;-\;\lambda E_{v},\\ {\dot{I}}_{a}\;=\;\lambda E_{a}-\alpha I_{a},\\ {\dot{I}}_{v}\;=\;\lambda E_{v}-\alpha I_{v}. \end{array}\right.$$

Avirulent and virulent nematodes compete for healthy plant roots *H^X^* in the following way: avirulent nematodes *P*_a_ can infect susceptible plants ( $\varepsilon_{a}^{S}\;=\;1$ ) but are unable to infect resistant plants ( $\varepsilon_{a}^{R}\;=\;0$ ), while virulent nematodes *P*_v_ are able to infect both resistant ( $\varepsilon_{v}^{R}\;=\;1$ ) and susceptible plants ( $\varepsilon_{v}^{S}\;=\;1$ ). Importantly, virulent nematodes grow more slowly than avirulent ones because they suffer from fitness costs, at two levels: reduced reproduction (*w_r_*) (Castagnone‐Sereno et al., 2007; Djian‐Caporalino et al., 2011; Jarquin‐Barberena et al., 1991; Meher et al., 2009) and reduced infectiveness (*w_β_*) (Castagnone‐Sereno et al., 2007; Castagnone‐Sereno, Mulet, & Iachia, 2015), the latter cost being weaker and more variable among nematode strains. We considered that there was no additional fitness cost (also called ''residual effect'') on resistant plants. Indeed, we conducted statistical tests and found no significant differences in terms of fitness costs when virulent nematodes grew on resistant *Mi‐1* or susceptible tomato plants (Castagnone‐Sereno et al., 2007). Furthermore, we assumed that a fraction *δ* of avirulent nematode offspring are virulent (Castagnone‐Sereno, Wajnberg, Bongiovanni, Leroy, & Dalmasso, 1994), due to mutation and/or epigenetic mechanisms. Following laboratory evidence showing that virulence is a stable character in resistance‐breaking nematode populations (Castagnone‐Sereno, Bongiovanni, & Dalmasso, 1993), we also assumed that, once acquired, virulence could not be lost by the virulent lineage. We can thus characterize a resistance gene and its susceptibility to resistance breakdowns with a set of three genetic parameters: the two fitness costs associated with nematode virulence (*w_β_* and *w_r_*) and the proportion of virulent variants in the nematode offspring (*δ*).

The initial conditions of the full multi‐seasonal model were set to *H^X^* (0) = *H*_0_, the initial root biomass of newly planted individuals, *P*_a_ = (1 – *p*_v_)*P*_0_ and *P*_v_ = *p*_v_ *P*_0_, where *P*_0_ refers to the initial nematode density in the soil and *p*_v_ to the initial proportion of virulent nematodes in the soil. Initial values of *I*_a_, *E*_a_, *I*_v_ and *E*_v_ were set to 0 because plants were assumed to be healthy at the time they were planted.

At the end of each cropping season, plants are removed. At the beginning of the next cropping season, healthy and infected roots are thus reset to their initial values, *H*_0_ and 0, respectively. Nematode densities *P*_a_ and *P*_v_ are set to their value at the end of the previous cropping season, multiplied by a survival probability *φ*. The full model of plant‐nematode interactions over multiple cropping seasons is therefore a hybrid model, with a continuous part to describe the nematode infection dynamics during a cropping season of length *τ*, and a discrete part to describe nematode survival between seasons (Mailleret et al., 2012; Mailleret & Lemesle, 2009). Note that parameter *φ* and the between‐season period can accommodate nonhost or poor‐host winter crops, such as salads or sorghum that are often used in combination with tomatoes. The sole requirement is that such winter crops do not differentially select for avirulent or virulent nematodes, which is what available evidence suggests (Djian‐Caporalino, personal communication).

For simulations and numerical investigations, models (Equations1 and 2) were implemented using the R software, version 3.4.4 (https://www.r‐project.org), and ordinary differential equations were solved with the deSolve R package (https://CRAN.R‐project.org/package=deSolve). We also analysed the existence and stability of the nematode‐free stationary solution and computed the season‐to‐season basic reproduction numbers *R*_0_ for avirulent and virulent nematodes (Mailleret et al., 2012). *R*_0_ computations are detailed in Appendix S1.

### 2.3 Model parameterisation

**FIGURE 2:**
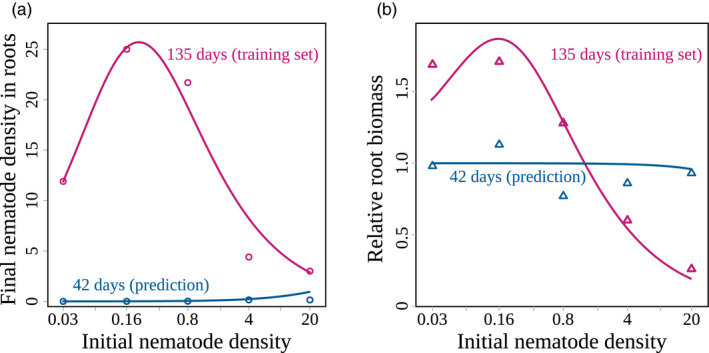
Fit of the model to experimental data over one cropping season (Ehwaeti et al., 1998). (a) Final density of nematodes in the roots and (b) relative biomass after 42 (in blue) and 135 days (in magenta), as functions of the initial density of nematodes in the soil (log scale). Model outputs are shown as solid curves, circles and triangles represent experimental measurements

Most parameter values could be set from published estimates in the literature (Table 1). No data were available for three parameters: the infection rate (*β*), the conversion factor between root biomass and density of feeding sites (*x*) and the plant growth scaling factor (*k*). Their values were thus estimated by fitting model (Equation 1) to an experimental data set reporting the final nematode density in plant roots as a function of initial nematode density in the soil (Ehwaeti et al., 1998). Specifically, avirulent *M. incognita* nematodes were inoculated at controlled densities in the soil, then tomato plants (cv *Moneymaker*) were planted and the nematode density in the root system was measured after 42 days and 135 days of cultivation. The relative root biomass (*i.e*. root biomass divided by the control root biomass with no nematode) was also measured. Both measurements after 135 days were used to fit our model, and the measurements at day 42 were compared with predicted values to assess model validity (Figure 2). More details are available in Appendix S2.

Parameters characterizing virulent nematodes, that is the fitness costs, were selected from data on the *Mi‐1* resistant tomato (Castagnone‐Sereno et al.,  2007). All parameters are summarized in Table 1.

### 2.4 Performance of resistance deployment strategies

We considered several resistance deployment strategies: the two “pure” resistant‐only and susceptible‐only strategies, consisting in planting one crop type all the time; periodic rotation strategies, alternating resistant and susceptible plants according to a repeated pattern; and unconstrained strategies, that is arbitrary sequences of susceptible and resistant plants.

The performance of each strategy was quantified with the “healthy root density”( $\bar{\text{HRD}}$ ), a proxy of crop yield defined as the mean of the integral of healthy plant root densities over the *n* cropping seasons:

(3) $$\bar{\text{HRD}}\;=\;\frac{1}{n}\sum_{i=1}^{n}\int_{i\text{th}\;\text{season}}H^{X}\left(t\right)\text{dt}$$

This quantity is similar to the healthy leaf area duration (HAD), the integral of healthy green canopy area during a growing season, used by many authors for airborne pathogens (Elderfield, Lopez‐Ruiz, van den Bosch, & Cunniffe, 2018; Gooding, Dimmock, France, & Jones, 2000; Lo Iacono, van den Bosch, & Paveley, 2012; Papaïx et al., 2018; Van den Bosch & Gilligan, 2003; Waggoner & Berger, 1987).

The durability of resistance was then defined as the number of consecutive seasons the resistant crop can be planted without losing more than 1% of crop yield ( $\bar{\text{HRD}}$ ), compared with the first year the resistance is used. This definition is close to the “usefulness time” used in Van den Bosch and Gilligan (2008), that is the number of seasons until the yield drops under a preset threshold. Such a metric helps assess the severity of the resistance‐breaking problem at hand.

### 2.5 Acceptable, efficient, and optimal strategies

**FIGURE 3:**
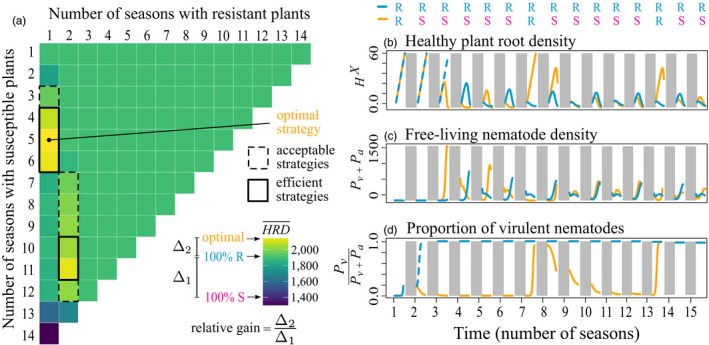
(a) Performance ( $\bar{\text{HRD}}$ , colour scale) of all periodic rotation strategies, according to their number of seasons of resistant (in columns) and susceptible (in rows) plants, over a 15‐season time horizon; performance of the susceptible‐only, resistant‐only and optimal strategies are indicated on the colour scale. The relative gain is defined as the gain in performance obtained by shifting from the resistant‐only to another strategy, relative to the gain in performance obtained by shifting from the susceptible‐only to the resistant‐only strategy. The optimal periodic rotation strategy 1*R* + 5*S* is identified by a black dot. Dotted‐ and plain‐line framed strategies represent acceptable periodic rotation strategies (relative gain > 0) and efficient periodic rotation strategies (relative gain > 50% of the optimal relative gain). (b–d) Graphical representation of two strategies: the resistant‐only strategy (in blue) and the 1*R* + 5*S* periodic strategy (in gold), which is optimal over a 15‐season time horizon; shaded areas correspond to the inbetween seasons. Default parameter values were used (Table 1)

In order to quantify the benefit of each resistance deployment strategy (or lack thereof), we computed its relative gain. It is defined as the gain in healthy root density ( $\bar{\text{HRD}}$ ) that the strategy provides over the resistant‐only strategy, normalized by the gain that the resistant‐only strategy provides over the susceptible‐only strategy. This is illustrated in Figure 3. For a given number of cropping seasons, that is a given time horizon, a positive relative gain indicates that the strategy outperforms the resistant‐only strategy, whereas a negative value indicates that the resistant‐only strategy is better. By definition, the relative gain of the resistant‐only strategy is equal to zero. This metric is useful to determine whether, and how much, rotation strategies are an improved way to deploy plant resistance. Moreover, it allows comparisons across parameter values and epidemic situations (see also Fabre, Rousseau, Mailleret, and Moury (2015)).

Based on this metric, we identified three types of strategies (Figure 3). The optimal strategy is defined as the strategy that maximizes the crop yield proxy $\bar{\text{HRD}}$ (Equation 3) and thus the relative gain. Efficient strategies are defined as all strategies that provide a relative gain at least 50% as high as the optimal strategy. Last, acceptable strategies are all strategies with a positive relative gain, that is strategies that outperform (even modestly) the resistant‐only strategy. In what follows, the main topic of interest will be to improve the efficacy of resistant plant‐based nematode control strategies. Therefore, we will essentially concentrate on efficient and optimal deployment strategies.

In order to identify optimal periodic strategies, we computed all periodic rotation strategies, beginning with resistant crops and alternating *m* and *p* seasons of resistant and susceptible plants, respectively. We denoted by *mR* + *pS* these periodic strategies. As an example, Figure 3a displays the healthy root density ( $\bar{\text{HRD}}$ ) of all periodic rotation strategies over a 15‐season time horizon. The optimal periodic strategy is 1*R* + 5*S*, which corresponds to 1 season of resistant plants followed by 5 seasons of susceptible plants, and so on. A graphical representation in Figure 3b–d displays the nematode and plant root dynamics over time. We also identified unconstrained optimal strategies by using a genetic algorithm implemented through the genalg R package (https://CRAN.R‐project.org/package=genalg). A chromosome in the genetic algorithm represented a full sequence of susceptible (*S*, coded as 0) or resistant (*R*, coded as 1) plants over the time horizon considered. The population of chromosomes (population size: 200) was initiated with random chromosomes. At each generation, the best 20% of chromosomes (according to our yield proxy) were retained to form the next generation, with mutation occurring at rate 0.01 (default parameter values of the package). The algorithm was run for 50 generations, for each time horizon. Convergence generally occurred in no more than 10 generations. The best chromosomes in the final generation were used to determine the set of optimal unconstrained strategies. We determined optimal strategies for time horizons between 1 and 30 cropping seasons. We also reported the corresponding ratios of resistant plants, that is the number of seasons with resistant plants divided by the total number of seasons.

### 2.6 Parameter exploration and epidemic scenarios

To assess the impact of parameter values, we performed a global sensitivity analysis (Saltelli et al., 2008) on the healthy root density $\bar{\text{HRD}}$ (Equation 3), the yield proxy which quantifies the performance of the resistance deployment strategies. We used the multi‐seasonal model (Equation 2) and simulated the optimal periodic rotation strategy over a 15‐season time horizon. We varied all parameter values by ± 30% (default values given in Table 1), except for the initial nematode density in the soil *P*_0_, for which larger variations were tested, in line with Ehwaeti et al. (1998). More details are available in Appendix S3. The most influential parameters were found to be the nematode reproduction rate *r*, the infection rate *β*, the nematode mortality rate in the soil *η* and the nematode mortality rate in the root *α*, four epidemiological parameters which explained more than 80% of the total $\bar{\text{HRD}}$ variability (Figure S3). By varying these parameters around their default value, we defined four epidemic scenarios, corresponding to different levels of epidemic severity, from Low to Extreme (Table 2).

**TABLE 2:** Definition of the four epidemic scenarios based on the four most influential parameters: nematode reproduction rate (*r*), infection rate (*β*), nematode mortality in the soil (*η*) and in the roots (*α*)

Scenario	*β*	*r*	*α*	*η*
Low	−30%	−30%	+30%	+30%
Medium	–	–	–	–
High	+30%	+30%	–	–
Extreme	+30%	+30%	−30%	−30%

Note

Default parameter values (–) or default values ± 30% were used (all values in Table 1).

Furthermore, we analysed with particular attention the influence of the genetic parameters (fitness costs *w_β_*, *w_r_* and proportion of virulent offspring *δ*), possibly in combination with the epidemic scenarios, on the nature and relative gain of optimal rotation strategies. Specifically, we sought to determine when optimal rotation strategies could outperform the usual resistant‐only strategy and to what extent crop yield could be increased by using such rotation strategies.

### 2.7 Robustness to parameter uncertainty

Finally, we evaluated the robustness of our results to determine to what extent optimal periodic strategies would remain effective if biological parameters were not known with perfect precision. For the Medium, High and Extreme epidemic scenarios defined in Table 2, the optimal periodic strategy was computed over a 15‐season time horizon and its performance was tested against ± 10%, 20% and 30% variations of all parameters except the initial nematode density in the soil *P*_0_, for which larger variations were tested (Table 1). In contrast with the analysis focusing on the impact of the genetic parameters, the periodic strategy was not computed afresh when the parameters varied. For each epidemic scenario, we explored the parameter space using a fractional factorial design containing 2,187 parameter combinations. The design was obtained using the planor R package (https://CRAN.R‐project.org/package=planor).

## 3 RESULTS

### 3.1 Optimal and efficient deployment strategies

**FIGURE 4:**
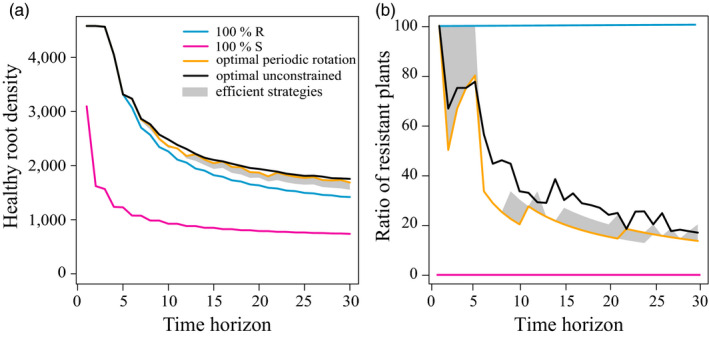
(a) Healthy root density ( $\bar{\text{HRD}}$ ) and (b) ratio of resistant plants as functions of the time horizon, for different deployment strategies: susceptible‐only (magenta), resistant‐only (blue), efficient periodic rotations (grey area), optimal periodic rotation (gold) and optimal unconstrained (black). Different unconstrained optimal strategies (yielding the same $\bar{\text{HRD}}$ ) were identified, so the ratio of resistant plants is represented in panel (b) by its average value (the ratio range is represented in Figure S2). Default parameter values were used (Table 1)

The performances (crop yield proxy $\bar{\text{HRD}}$ ) of pure, optimal and efficient deployment strategies are shown in Figure 4a, for different time horizons and the default parameters. As expected, the resistant‐only, efficient and optimal strategies outperformed the susceptible‐only strategy, since the deployment of resistance prevents infection by avirulent nematodes. However, for these strategies, the crop yield proxy decreased with the time horizon. This is also expected, as the deployment of resistance also causes virulent nematodes to appear and take over the nematode population.

For up to five years of cultivation, the resistant‐only strategy performed as well as any optimal deployment strategy, but over longer time horizons, it could be significantly outperformed. For instance, over 15 cropping seasons, the healthy root density was around 2,044 UR.day for optimal strategies, while it had dropped to 1,822 UR.day for a pure resistant‐only strategy (Figure 4a). By definition, efficient periodic rotations performed better than the resistant‐only strategy and were worse than but close to the optimal periodic rotation. Interestingly, for all time horizons considered (up to 30 years), the optimal periodic and the unconstrained strategies had almost identical performances. This indicates that periodic rotations are almost optimal in this system.

The deployment of a pure resistant‐only strategy is thus reasonable for at most five years in this cropping system. Beyond that, the optimal strategy generally was to alternate one season of resistant plants with a few seasons of susceptible plants, as shown for instance in Figure 3 for a 15‐season time horizon. This optimal strategy ensures that virulent nematodes remain sufficiently rare in the soil, sustaining the efficiency of resistant plants, which severely reduce the avirulent nematode population. Other periodic rotations outperformed the resistant‐only strategy. Yet, while there was generally one single optimal periodic rotation strategy for a given time horizon and parameter set, there were only a few acceptable and even less efficient rotation strategies (*e.g*. 10 acceptable and 5 efficient strategies out of 105 periodic rotation strategies; Figure 3a).

For a given time horizon, the average ratio of resistant plants characterizing the unconstrained optimal strategy was generally higher than for the optimal periodic rotation strategy; for acceptable and efficient periodic rotations, the ratio also tended to be slightly higher than for the optimal periodic rotation (Figure 4b). For instance, over a 15‐season time horizon, the genetic algorithm identified 11 equivalent solutions and the ratio of resistant plants deployed was on average 30%. For the optimal periodic strategy, it was only 20% and for acceptable and efficient periodic rotation strategies, it ranged between 20% and 27%. Unconstrained optimal strategies identified by the genetic algorithm were actually fairly similar to optimal periodic rotations in terms of structure, except that more resistant plants were used in the final seasons, explaining the higher ratio of resistant plants in unconstrained strategies.

### 3.2 Influence of fitness costs

**FIGURE 5:**
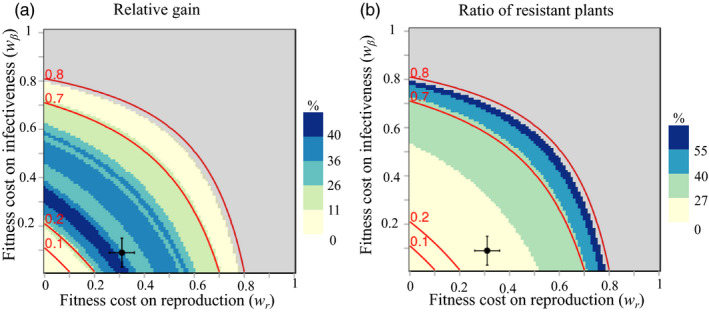
(a) Relative gain and (b) ratio of resistant plants as functions of the two fitness costs, for optimal periodic strategies computed over a 15‐season time horizon. The grey area corresponds to fitness costs for which the resistance was fully durable over the 15‐season time horizon. Level curves in red represent different values of the effective fitness cost *w* ^*^ defined in (Equation 4). The black dot and the error bars indicate the default fitness costs and their standard deviations (Castagnone‐Sereno et al., 2007)

We computed the optimal periodic rotation strategies as functions of the two fitness costs on infectiveness (*w_β_*) and reproduction (*w_r_*), to explore their effects on the two metrics defined above: the relative gain brought about by optimal periodic rotations and the resistance durability. Results are displayed in Figure 5a.

The area where resistance was durable for (at least) the entire 15‐season time horizon is found in the upper right part of the figure. This area corresponds to R‐genes associated with very strong fitness costs of one or the other kind (*w_β_* ≥ 0.8 or *w_r_* ≥ 0.8). This means that rotation was unnecessary in such conditions, at least for the time horizon considered. For lower fitness costs, resistance was not durable and thus the use of optimal periodic rotation strategies produced a better crop yield than the resistant‐only strategy (positive relative gain).

The relative gain was fairly high, except in two cases. On the one hand, when resistance breaking entailed low fitness costs (*w_β_* or *w_r_* ≤ 0.12), the relative gain was almost zero. This is not surprising since for such low fitness costs, virulent nematodes cannot be prevented from overturning the nematode population, even with rotation strategies, as they develop quite well on both resistant and susceptible plants. Cropping resistant plants is then useless and does not provide any increase in yield. On the other hand, R‐genes associated with high fitness costs (*w_β_* or *w_r_* ≥ 0.7) provided a relative gain of less than 10%. For such fitness costs, resistance durability was in fact quite high (12–14 seasons). Therefore, the resistant‐only strategy was quite efficient and the additional yield provided by periodic rotations is minimal.

Significant relative gains are thus observed for R‐genes inducing medium fitness costs in virulent nematodes. Relative gains can in this case reach values up to 50%. Interestingly, in the literature, the fitness cost on reproduction *w_r_* is estimated between 0.26 and 0.36 and the fitness cost on infectiveness *w_β_* between 0.03 and 0.15, for the susceptible Saint Pierre tomato cultivar (Castagnone‐Sereno et al., 2007). For such realistic fitness cost values, the expected relative gain that could be realized by switching from a resistant‐only strategy to an optimal periodic rotation would be between 26% and 43% with a relative gain equal to 28% for the default values parameter values.

The ratio of resistant plants deployed in the optimal periodic rotation strategies in order to achieve such relative gain values were remarkably low, lying between 13% and 27% (Figure 5b). For the default parameter values, the ratio of resistant plants was 20%. The ratio of resistant plants used in the optimal rotation strategies increased with the values of the fitness costs.

Interestingly, Figure 5 shows that the fitness cost distribution between infectiveness and reproduction is important for crop yield. Indeed, even though the two fitness costs had perfectly symmetrical effects, the level curves of both the relative gain and the ratio of resistant plants were markedly concave. Therefore, a balanced distribution of fitness costs (*e.g. w_β_ = w_r_ = *0.4) could lead to a situation where resistance was not durable, while an uneven distribution (*e.g. w_r_ = *0.8, *w_β_ = *0) could lead to a durable situation. The two fitness costs thus did not act in an additive manner and interacted negatively. The derivation of the multi‐season basic reproduction number *R*_0_ of virulent nematodes revealed that it depended only on the product (1 − *w_β_*) (1 − *w_r_*) (Appendix S1). We hence defined an “effective” fitness cost as:

(4) $$w^{*}\;=\;1\;-\;\left(1\;-\;w_{\beta }\right)\left(1-w_{r}\right)\;=\;w_{\beta }\;+\;w_{r}\;-\;w_{\beta }w_{r},$$

whose level curves perfectly reflected the level curves of the relative gain and ratio of resistant plants (Figure 5). The performance of resistance‐based strategies therefore appeared to be entirely determined by this quantity.

In the following, we thus present results in terms of this effective fitness cost *w* ^*^.

### 3.3 Interplay between epidemic scenarios and genetic parameters

**FIGURE 6:**
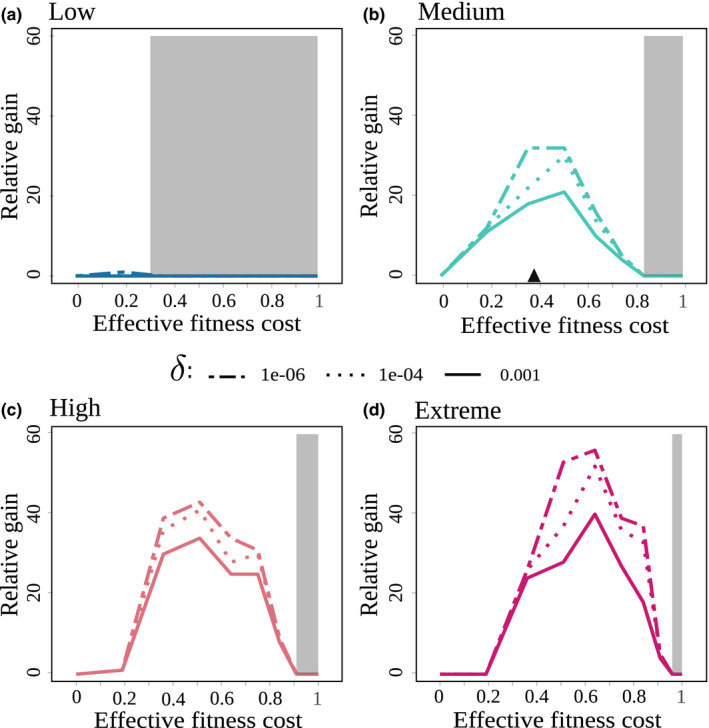
FIGUREGraphical representation of the relative gain for a 15‐season time horizon for the four epidemic scenarios (a–d) defined in Table 2, as a function of the effective fitness cost (w*) and the fraction of virulent offspring (δ). The default effective fitness cost *w* ^*^ = 0.37 is represented by the black triangle (Castagnone‐Sereno et al., 2007). Grey areas represent the values of *w* ^*^ for which the resistance was durable over the 15‐season time horizon

We studied the influence of the genetic parameters in interaction with the epidemic scenarios on the relative gain and durability. Figure 6 shows the relative gain obtained for a 15‐season time horizon as a function of the effective fitness cost (*w* ^*^), for different values of the fraction of virulent offspring (*δ*) and the four epidemic scenarios. Parameter ranges ensuring resistance durability over the 15‐season time horizon were identified (grey areas). *δ* had no effect on durability according to our definition. Indeed, when only resistant plants were deployed, avirulent nematodes could not reproduce. The resistance was durable as the effective fitness cost *w* ^*^ overshot a given threshold, which strongly increased with the severity of the epidemic scenario. For instance, for low epidemic severity, R‐genes associated with effective fitness costs between 0.3 and 1 were durable (Figure 6a), while in the Extreme scenario, they were durable only for fitness costs larger than 0.95 (Figure 6d).

The relative gain varied significantly according to the genetic parameters and epidemic scenarios, except for the Low epidemic scenario where it remained close to zero (Figure 6a). In this case, nematode infestation remained very low so that the resistant‐only strategy actually provided very good control. The relative gain increased with epidemic severity and decreased with the fraction of virulent offspring *δ*. The best gains were found for R‐genes associated with medium to high effective fitness costs (between 0.4–0.65). For example, an extreme severity combined with a low fraction of virulent offspring *δ* = 10^−6^ and a fitness cost *w* ^*^ = 0.65 yielded a relative gain of up to 58% (Figure 6d). Hence, epidemic severity tended to increase the advantages of cultivar rotations over the resistant‐only strategy.

### 3.4 Robustness of deployment strategies

**FIGURE 7:**
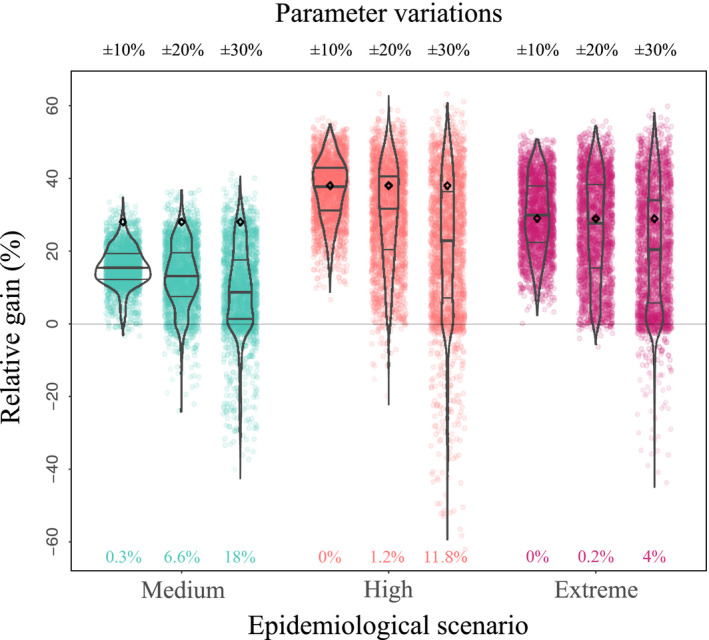
Robustness of the relative gain to variations in model parameters, for three epidemic scenarios (Medium, High, Extreme) and three levels of parameter variations (±10%, 20%, 30% and variations in the initial nematode density as reported in Table 1). For each scenario, the optimal periodic strategy, computed for a 15‐season time horizon and default parameters values (Tables 1 and 2) was applied. The relative gain of this strategy was computed for all parameter combinations within each level of parameter variations. In each case, the relative gains obtained for the default parameter values (black diamond) and for the different parameter combinations (2,187 coloured dots) were plotted. Violin plots were drawn to help quantification, with horizontal bars indicating the median (thick line), first and third quartiles (thin lines). The percentages provided on the bottom line correspond to the fraction of parameter combinations which yield nonpositive relative gains, that is for which the optimal strategy would not be acceptable (Figure 3)

Finally, we evaluated the robustness of the optimal periodic rotation strategies by testing their efficacy against variations in parameter values. Figure 7 represents the relative gain when deploying the optimal periodic strategy, computed over a 15‐season time horizon and for the default parameters corresponding to the three epidemic scenarios, in the face of increasing levels of parameter variations. Such variations can effectively render the computed rotation strategies suboptimal.

In a large majority of cases, the relative gain remained positive, although it declined, as expected, with the level of parameter variations. In the Medium epidemic scenario, most parameter combinations decreased the relative gain below the 28% gain predicted for default parameter values (black diamond in Figure 7). The median relative gain was between 8% and 20%, depending on the level of parameter variations. Note however that some parameter combinations actually resulted in higher‐than‐expected relative gains. The situation was even more favourable in the High and Extreme epidemic scenarios, for which the decline in relative gain was less pronounced. In addition, a significant fraction of parameter combinations caused an increase in the relative performance of the optimal rotation strategy (Figure 7).

Parameter combinations causing the rotation strategy to become nonacceptable, that is for which the strategy failed to provide a positive relative gain, were rare overall, especially for the most severe epidemic scenarios. At most, these combinations represented 18% of all combinations (for ± 30% variations in the Medium epidemic scenario). The optimal strategy 1*R* + 5*S*, even in the face of important parameter uncertainty, thus retained higher performance than the resistant‐only strategy in more than 82% of the cases tested. In that sense, the relative performance of the optimal periodic strategy was globally very robust to parameter changes.

## 4 DISCUSSION

### 4.1 Crop rotation is an efficient strategy

The present study was based on a new model of plant‐nematode interactions parameterized from the literature and fitted to experimental data, so as to be representative of the tomato/root‐knot nematode system. As a key result, we found that alternating susceptible and resistant plant cultivars in time can help limit the proportion of virulent individuals in nematode populations and thereby reduce crop loss substantially. According to our simulations, relative gains as high as 40% can be achieved, compared with the baseline strategy of deploying only resistant plants, over time horizons of 15 years or more.

The relative gain achievable with optimal crop rotations was found to be greatest for High or Extreme epidemic scenarios, that is for high epidemic severities. The latter result echoes previous findings on the influence of epidemic intensity on resistance durability in the context of spatial mixtures (Fabre et al., 2012; Van den Bosch & Gilligan, 2003). The gain also increased, to a smaller extent, if the fraction of virulent offspring in avirulent egg‐clutches is smaller, and if the culture is sustained over longer temporal horizons. Remarkably, the relative gain obtained from virulence costs similar to those estimated for the *Mi‐1*resistant gene is close to the maximum achievable gain value (Figure 5a), suggesting that crop rotation is a particularly promising strategy when deploying *Mi‐1*cultivars.

We also found that periodic crop rotation strategies are almost as effective as free (unconstrained) alternation strategies. This result has considerable importance, since periodic rotation patterns are in real‐world applications much easier for crop growers to implement than complicated unconstrained sequences.

Few recent theoretical studies have considered the deployment of different cultivars over time. One is Rimbaud et al. (2018), that compared four resistance deployment strategies of major resistance genes: mosaics, mixtures, rotations and pyramiding, to manage cereal rust fungi in agricultural landscapes durably. They found cultivar rotation to be the most efficient in the long‐term, once every R‐genes had been overcome. In a study of plant virus epidemic control by mixing resistant and susceptible cultivars in space and time, Fabre et al. (2015) identified that in more than 20% of the scenarios considered, optimal strategies involved cultivar rotation at the landscape scale. Studies are even scarcer regarding root‐knot nematodes, for which the literature on cultivar rotation is essentially experimental. For these low‐dispersing soil‐borne pests, data support our modelling predictions in suggesting that rotations are an effective way to reduce yield losses and to delay outbreaks (McSorley, 2011; Miller et al., 2006; Tzortzakakis, Phillips, & Trudgill, 2000). For instance, Djian‐Caporalino et al. (2014) experimentally compared the performance of several strategies to control root‐knot nematodes in vegetable cropping systems, including rotations of two major R‐genes in pepper cultivars, over 3 years. They reported that cultivar rotation can improve epidemic control and resistance durability. Another study by Talavera et al. (2009) on root‐knot nematode management compared the effects of four crop rotations between resistant and susceptible tomato plants in a three‐year field experiment. Regarding crop yield and durability, this study showed that the best strategy consisted in growing two resistant cultivars, followed by one susceptible cultivar. This is strikingly consistent with our modelling predictions, since we found that the yield‐maximizing strategy, over a three‐season temporal horizon, is 2*R* + 1*S* (Figure 4b). Our modelling results further indicate that the performance of crop rotations for root‐knot nematode control would be even more pronounced over longer time horizons.

### 4.2 Crop rotation (usually) requires low ratios of resistant plants

Interestingly, the optimal rotation strategies identified in this study were characterized by relatively low ratios of resistant plants, as soon as the temporal horizon exceeded seven cropping seasons (Figure 5b). Since avirulent nematodes thrive on susceptible plants, low ratios of resistant plants are expected to increase crop loss, especially in the short‐term. However, in the longer term, low ratios limit selection for virulent variants, thus prolongating the efficacy of resistant plants when those are deployed. For root‐knot nematodes, it appears that the relatively fast within‐season epidemiological dynamics sets the optimal balance between the two effects at a low ratio of resistant plants. Our results are consistent with Van den Bosch and Gilligan (2003), who showed that, in many instances, low ratios allowed to make the most of resistance, by reducing the selection pressure for virulent pathogens and promoting resistance durability.

Interestingly, studies of spatial deployment strategies tend to report higher optimal ratios of resistant plants. Fabre et al. (2012), working on plant resistance to viruses, demonstrated that optimal ratios were frequently over 50%. For instance, for low fitness costs, the ratio ranged between 50% and 70%, depending on the epidemic profile. Regarding phytopathogenic fungi, Papaïx et al. (2014) also found that high ratios combined with low levels of variety aggregation provided optimal control of the fungi in agricultural landscapes. Therefore, the selection pressure in favour of virulent variants seems to be lower when mixing resistant and susceptible cultivars in space compared with alternating them over time.

It should be remarked that low ratios of resistant plants are in total contrast with the currently dominant agricultural practices, based on the regular cropping of tomato cultivars bearing the same *Mi‐1* resistance gene. Indeed, growing resistant tomatoes is the best strategy over a single cropping season. However, be it in the field or in experimental studies, such resistant‐only strategies often fail, and virulent root‐knot nematodes overcoming resistance have been observed in most tomato growing areas worldwide (Seid et al., 2015). More specifically, experimental findings have shown that three consecutive cropping seasons of the *Mi‐1* gene in tomatoes were enough for nematodes to overcome the resistance (Eddaoudi, Ammati, & Rammah, 1997; Verdejo‐Lucas et al., 2009). These findings are consistent with our results when fitness costs are not too severe, close to available experimental estimates (Castagnone‐Sereno et al., 2007; Djian‐Caporalino et al., 2011). The intense deployment of resistant cultivars is thus bound to cause boom and bust cycles in this system (Brown & Tellier, 2011). During the boom, crop yield increases rapidly thanks to the use of new resistant cultivar by growers and farmers. Nevertheless, it is followed by a bust, characterized by the rapid breakdown of the resistance by virulent variants and a drop in crop yield. The switch to a new cultivar, carrying a fresh resistance gene, then triggers a new cycle. To break this cycle and preserve the efficiency of resistance genes, which are scarce and valuable resources, cultivar rotations such as the ones proposed in this study are a feasible and sustainable alternative. However, convincing growers to switch from a short‐term to a long‐term perspective may be an issue. It would require close interactions between scientists and growers to co‐design acceptable resistance deployment strategies.

### 4.3 What makes a good resistance gene?

We investigated the effects of varying three mechanistic parameters characterizing how a resistance gene behaves with respect to resistance breaking by nematodes: the fitness cost it imposes on the infectivity of virulent nematodes (*w_β_*), the fitness cost it imposes on their reproduction (*w_r_*) and the frequency of virulence appearance in avirulent clutches (*δ*). Obviously, one would seek R‐genes that, when overcome, would generate high values of the first two parameters, and low values of the third, even though it may not necessarily be easy to evaluate.

Our results showed that the two fitness costs had interchangeable effects in shaping the population dynamics of virulent variants. However, the two costs interacted negatively, as the benefit of increasing one fitness cost was reduced when the other fitness cost is already high (Figure 5a). This original result implies that, when evaluating the potential of resistance genes to improve durability, breeders should seek and introgress R‐genes with maximal fitness cost on either one or the two components of the nematode life cycle (reproduction or infectivity), rather than a balanced distribution of the two types of costs. To help address the existence of two different types of fitness costs, a specificity of our model, we derived a simple formula to synthesize the two fitness costs into one effective fitness cost, according to which different resistance genes can be ranked in terms of their durability. Comparatively, the rate of production of virulent nematodes *δ* had virtually no impact on the durability of resistance genes.

Rotation strategies provided the largest relative benefits over the resistant‐only strategy for intermediate fitness costs. Such measurements are not always easily accessible in the literature, but this property seems to hold in a few other studies. For instance, a reinvestigation of the simulation data on plant virus epidemics obtained by Fabre et al. (2012) for high epidemic intensities showed that the best relative gains were obtained for intermediate fitness costs. Another study by Rousseau et al. (2019) showed that relative additional gains, provided by combining quantitative and qualitative resistances over qualitative resistances only, were most noticeable for intermediate fitness costs. In both studies, the reasons for this were similar to the present study: high fitness costs induced durable resistance so that the yield could only be marginally increased, whereas low costs induced poorly efficient resistance that did not benefit from an optimal deployement. R‐genes associated with intermediate fitness costs are thus the ones that could benefit the most from improvements in terms of deployment or cultivar genetic background.

### 4.4 Optimal rotations in practice

A major outcome of this work would be to recommend custom optimal resistance deployment strategies to crop growers, depending on the temporal horizon sought, but also on the epidemic context, the R‐genes to be deployed and on the agricultural practices that determine model parameter values. Indeed, even though optimal resistance deployment has been proven to be efficient, quite few periodic rotation strategies are actually “acceptable” and even less are “efficient” (Figure 3a). The pattern of the rotation, in particular the ratio of resistant plants, is critical. In addition, soil infestation and epidemiological or genetic parameters are particularly difficult to estimate and likely subject to considerable uncertainty. For instance, Djian‐Caporalino et al. (2011) found a large variability in the fitness costs on reproduction. To address this issue, we simulated the use of optimal periodic strategies, as computed for default parameter values, and investigated how their performance responded to parameter variations. We found that the relative gain was globally robust to parameter changes. Thus, optimal periodic rotations can outperform the resistant‐only strategy in terms of crop yield even if the relevant parameters are known imperfectly. Rotating susceptible and resistant cultivars is not necessarily a good idea. However, rotating wisely (optimally) can provide significant gains and is robust to parameter uncertainties, which is a very desirable property in practice.

There are still few studies that investigate the robustness of resistance deployment strategies, or more generally plant pathogen control methods. A similar analysis to parameter misspecification was conducted by Hyatt‐Twynam et al. (2017) to assess the performance of optimal strategies to control the spread of citrus canker in Florida, using one at a time epidemiological parameter changes. In the context of fungicide resistance management, Elderfield et al. (2018) found that mixtures always outperformed alternations when parameters varied, but not the deployment strategy. More such studies should arise to help bridge the gap between theoretical resistance deployment strategies and their implementation in the field.

Optimal strategies could feature high year‐to‐year variations in yield, which may not be economically viable for farmers. Taking advantage of the limited mobility of nematodes, this issue could be addressed by implementing asynchronous crop rotation strategies in different rows or plots, provided that contamination between those be carefully avoided. The seasonal yield variations in each row would average out, ensuring a more stable income for farmers while achieving the performance of the optimal rotation strategy.

Although nematode resistance in general can be conferred by single major genes or by combinations of genes or quantitative trait loci (QTL), root‐knot nematode resistance in solanaceous crops mainly relies upon single major dominant genes (Barbary, Djian‐Caporalino, Palloix & Castagnone‐Sereno, 2015). Currently, *Mi‐1* is the only resistance gene used in tomato cultivars, which makes this study particularly relevant for the tomato/root‐knot nematode pathosystem. In pepper, though, QTL have recently been identified (Barbary et al., 2016) and their efficiency was demonstrated in laboratory experiments (Barbary et al., 2014). As such they could be a promising source of resistance, alone or in combination with major R‐genes, which would deserve further modelling investigations along the lines of the present study. An ideal strategy would be to breed tomato varieties with unbreakable resistance. In the absence of such a silver bullet, rotation of varieties seems a viable alternative.

Provided sufficient parameter estimates are available, the model could effortlessly be extended to simulate rotations of tomato with other species (e.g. other Solanaceae or cucurbits), as is common in many cropping systems. The present model could also readily be extended to incorporate different R‐genes and serve as a basis to evaluate more complex resistance deployment strategies, involving rotations between susceptible and several resistant cultivars or species, including pyramided ones.

## CONFLICT OF INTEREST

None declared.

## Supporting information

Supplementary Material

## Data Availability

The data that support the findings of this study are available from the corresponding author upon reasonable request.
